# Supplementary material for: Legionella maintains host cell ubiquitin homeostasis by effectors with unique catalytic mechanisms
Source: Nat Commun. 2024 Jul 15;15:5953. doi: 10.1038/s41467-024-50311-2 (PMC11251166; doi:10.1038/s41467-024-50311-2)
Supplement: Supplementary file 1 — Supplementary Information [file 41467_2024_50311_MOESM1_ESM.pdf]

Legionella maintains host cell ubiquitin homeostasis by effectors with unique catalytic mechanisms

Jiaqi Fu[1*], Siying Li[1*], Hongxin Guan[2*], Chuang Li[3*], Yan-Bo Zhao[2*], Tao-Tao Chen[2], Wei Xian[4], Zhengrui Zhang[5], Yao Liu[3], Qingtian Guan[1], Jingting Wang[2], Qiuhua Lu[2], Lina Kang[2], Si-Ru Zheng[2], Jinyu Li[6], Shoujing Cao[6], Chittaranjan Das[5], Xiaoyun Liu[4¶], Lei Song[1¶], Songying Ouyang[2¶] and Zhao-Qing Luo[3¶]

Zhao-Qing Luo
Email: luoz@purdue.edu

Supplementary information

Supplementary Figures 1 to 11
Supplementary Tables 1 to 2

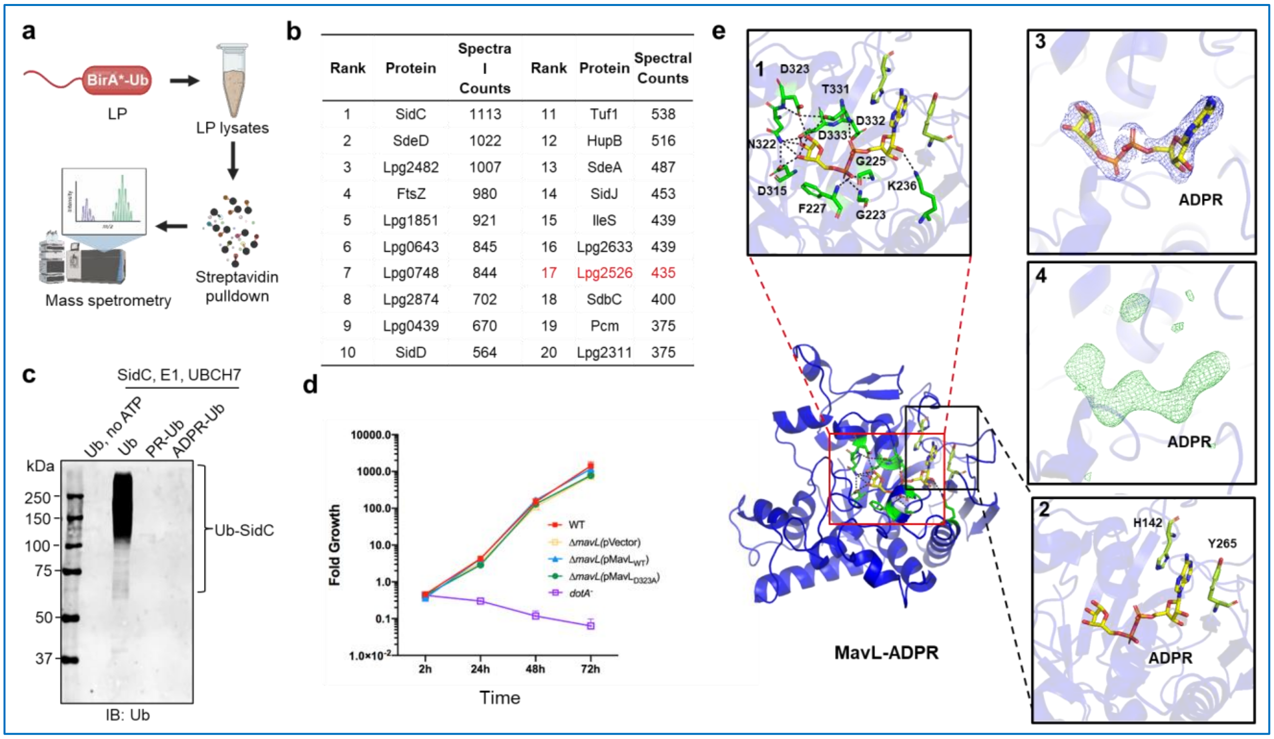

Supplementary Figure 1 MavL is a macrodomain protein capable of hydrolyzing ADPR-Ub

a-b. Identification of ubiquitin binding proteins in *L. pneumophila*. A diagram for the procedure, cell lysates of an *L. pneumophila* strain expressing the BirA*-Ub fusion was subjected to precipitation with streptavidin and the enriched proteins were identified by mass spectrometry analysis (a). The top 20 identified proteins (b).

c. PR-Ub and ADPR-Ub do not support canonical ubiquitination reaction. Equal amount of ubiquitin, PR-Ub or ADPR-Ub was used in ubiquitination reactions using SidC as the E3 ligase. A reaction receiving ubiquitin but no ATP was established as a control. Note that no ubiquitination occurs in reactions receiving PR-Ub or ADPR-Ub (4th and 5th lanes).

d. MavL is not essential for intracellular growth of *L. pneumophila.* Bacteria of the indicated strains were used to infect mouse bone marrow-derived macrophages at an MOI of 0.05 and the growth of the bacteria was determined at the indicated time points. Results shown were from two independent experiments each done in triplicate.

e. MavL recognizes ADPR through a unique macrodomain. Residues of MavL involved in the formation of the ADPR-binding pocket were shown as sticks, the hydrogen bond was indicated by dashed lines (1). H142 and R265 of MavL interacted with the adenine base of ADPR through π-π stacking (2). The 2Fo−Fc (blue) electron-density maps of

ADPR are contoured at the 1.0σ (3). The Fo−Fc (green) electron-density maps of ADPR are contoured at the 3.0σ (4).

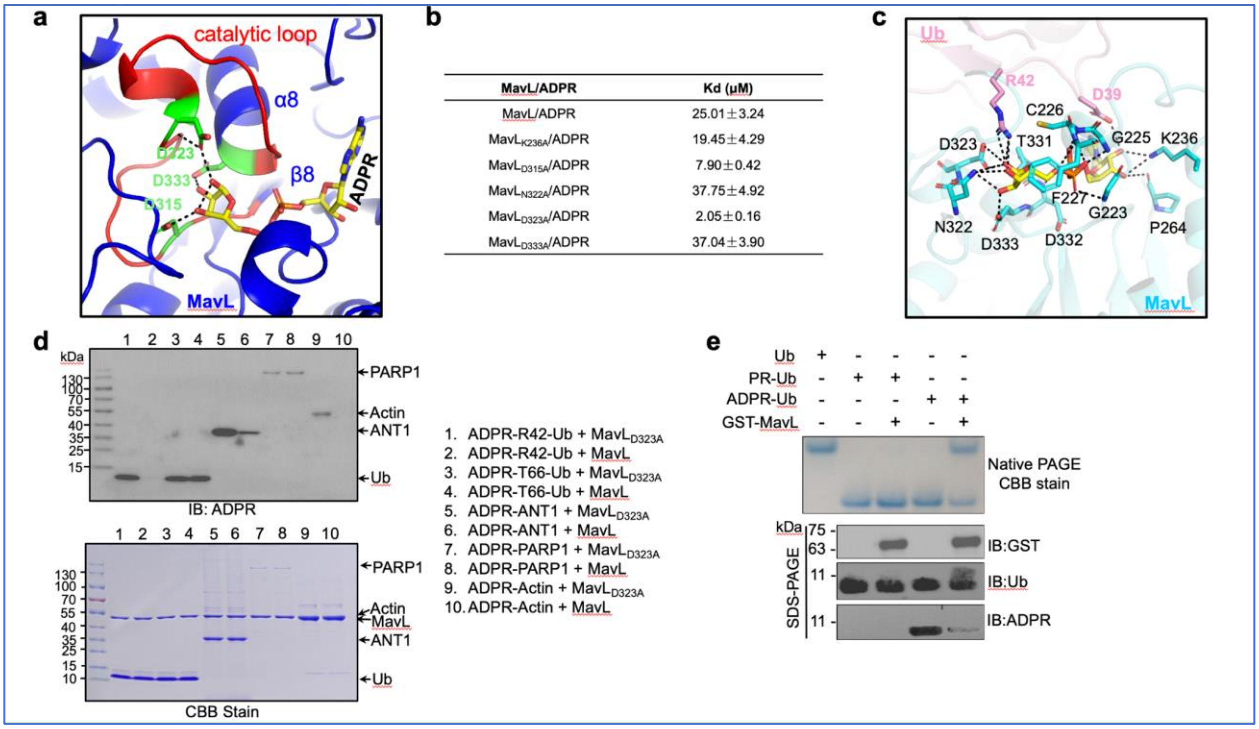

Supplementary Figure 2 The catalytic center of MavL and its substrate specificity

a. Residues D315, D323 and D333 shown as green sticks likely formed a catalytic loop. The loop between β8-α8 was shown in red.

b. Binding of ADPR to MavL and its mutants. The binding affinity was determined by ITC.

c. MavL and Ub interacted with ADPR through hydrogen bonding. The key residues involved in hydrogen bonding were shown as sticks.

d. MavL selectively hydrolyzed ADPR-Ub produced by SidEs. ADP-ribosylated proteins produced by the enzymes described in the main text were individually incubated with MavL for 1 h at 37°C and the removal of the ADPR moiety was detected by immunoblotting with an ADPR-specific antibody.

e. MavL cannot remove the phosphoribosyl moiety from PR-Ub. Recombinant MavL was mixed with PR-Ub or ADPR-Ub and the production of native ubiquitin was detected by native PAGE (upper). Reaction components were detected by immunoblotting with the appropriate antibodies (lower three panels).

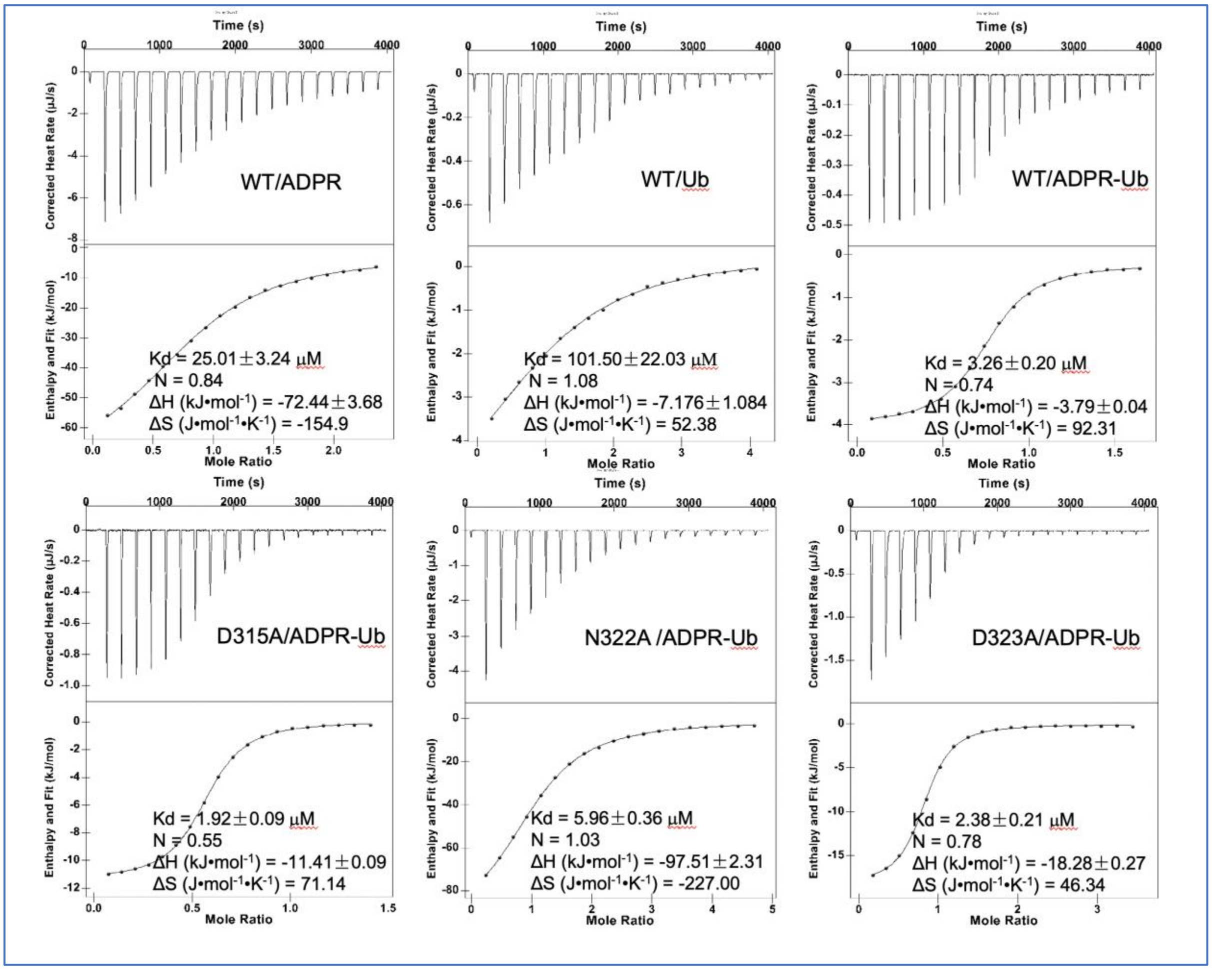

WT/ADPR
Kd = 25.01±3.24 μM
N = 0.84
ΔH (kJ•mol-1) = -72.44±3.68
ΔS (J•mol-1•K-1) = -154.9
WT/Ub
Kd = 101.50±22.03 μM
N = 1.08
ΔH (kJ•mol-1) = -7.176±1.084
ΔS (J•mol-1•K-1) = 52.38
WT/ADPR-Ub
Kd = 3.26±0.20 μM
N = 0.74
ΔH (kJ•mol-1) = -3.79±0.04
ΔS (J•mol-1•K-1) = 92.31
D315A/ADPR-Ub
Kd = 1.92±0.09 μM
N = 0.55
ΔH (kJ•mol-1) = -11.41±0.09
ΔS (J•mol-1•K-1) = 71.14
N322A /ADPR-Ub
Kd = 5.96±0.36 μM
N = 1.03
ΔH (kJ•mol-1) = -97.51±2.31
ΔS (J•mol-1•K-1) = -227.00
D323A/ADPR-Ub
Kd = 2.38±0.21 μM
N = 0.78
ΔH (kJ•mol-1) = -18.28±0.27
ΔS (J•mol-1•K-1) = 46.34
Time (s)
Corrected Heat Rate (μJ/s)
Enthalpy and Fit (kJ/mol)
Mole Ratio

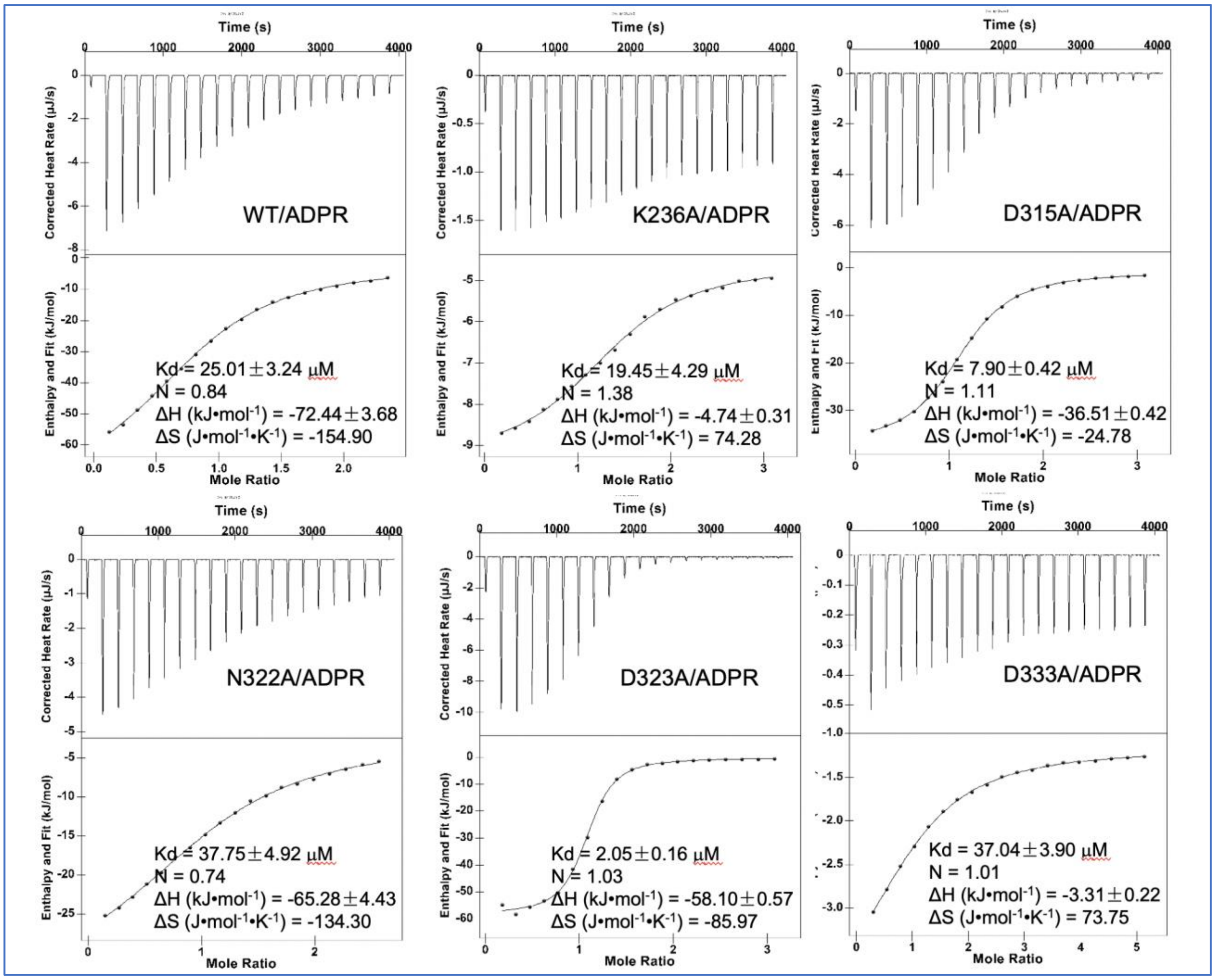

Supplementary Figures 3 and 4 The original results of the binding affinity of MavL and its mutants to ADPR-Ub or ADPR determined by ITC. These were the original data for results summarized in Fig. 1C and Fig. S2B, respectively. The binding affinity, stoichiometry and thermodynamic parameters are also shown.

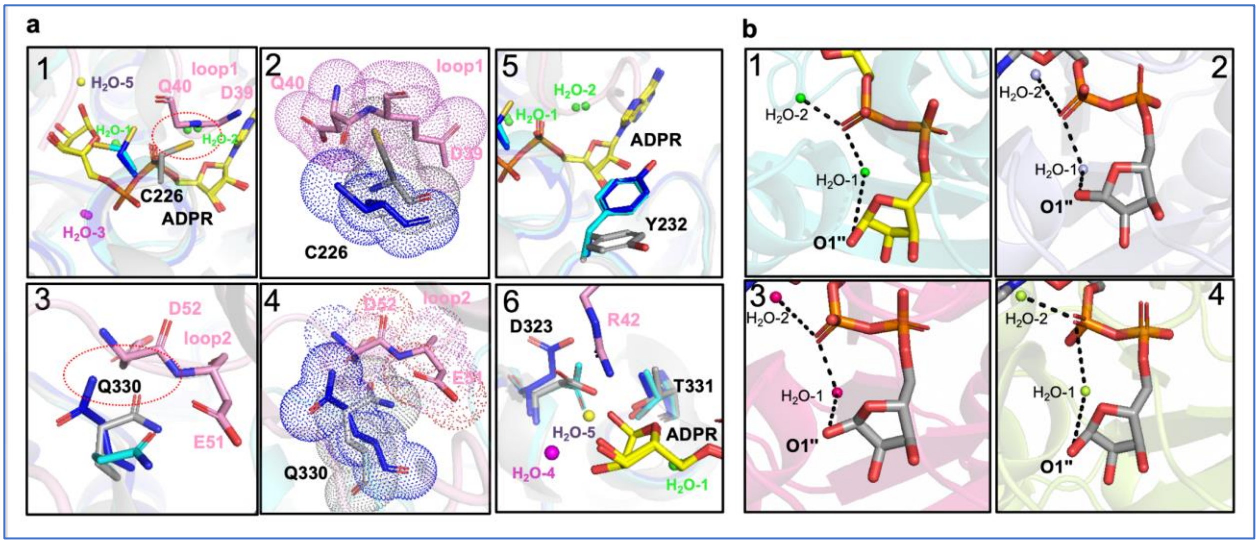

Supplementary Figure 5 Conformations of MavL in different statuses and its association with water molecules

a. Conformational changes of MavL in the catalytic pocket for the apo state, the ADPR binding state, and the substrate (ADPR-Ub) binding state. The C226 side chain of apo MavL has a significant steric hindrance (indicated by the red dashed square) with D39 and E40 in loop 1 of Ub. H_2O-1,2,3 is observed in both MavL-ADPR and MavL-ADPR-Ub . H_2O-4 is found only in MavL-ADPR-Ub, while H_2O-5 is found only in MavL-ADPR (1). The corresponding residues in (1) are shown as dots (2). The Q330 side chains of apo MavL and MavL-ADPR show significant steric hindrance (indicated by the red dashed square) to E51 and D52 in loop 2 of Ub (3). The corresponding residues in (3) are shown as dots (4). In contrast to the complexes, the Y232 side chain of apo MavL flips significantly to the side of the active pocket, making it more open (5). A unique water molecule (H_2O-4) was observed in MavL-ADPR, and the presence of this water molecule caused the side chain of D323 to fold in the opposite direction of ADPR (6).

b. H_2O-1 and H_2O-2 are also found in other macro domain ARHs. 1: MavL-ADPR (PDB: 8IPW), 2: The complex structure of *E. coli* Ymdb with ADPR (5CB3), 3: The complex structure of human MACROD1 with ADPR (6LH4), 4: The complex structure of *Trypanosoma brucei* with ADPR (5FSY).

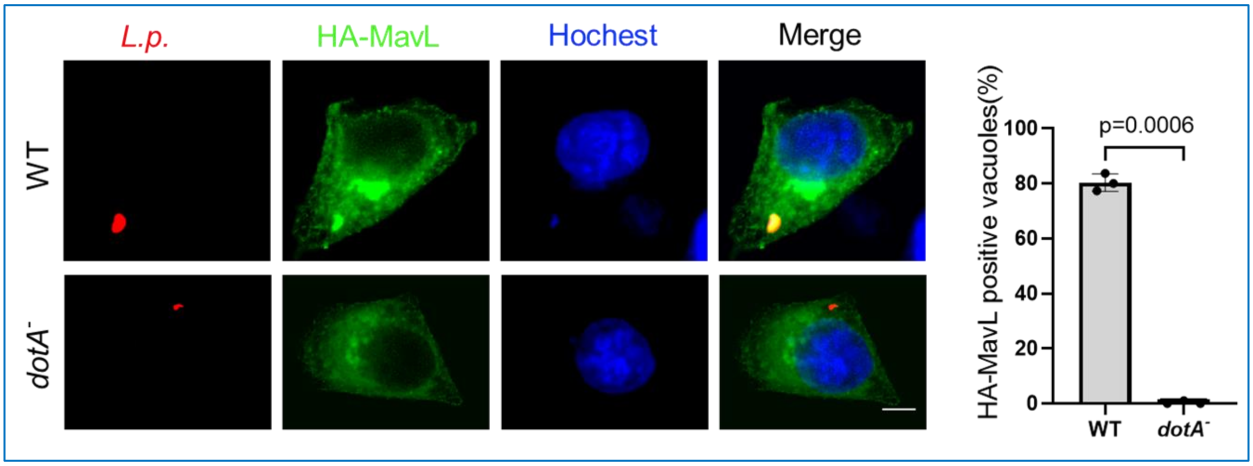

Supplementary Figure 6 Ectopically expressed MavL is associated with the Legionella phagosome. HEK293 cells transfected to express HA-MavL were infected with the indicated *L. pneumophila* strains for 2 h and the samples were stained with the appropriate antibodies. Shown are representative images of the bacterial vacuoles of the wild-type and the dotA mutant. Quantitation of the association was performed by counting at least 100 vacuoles for each sample. Results shown were from 3 independent experiments each done in triplicate.

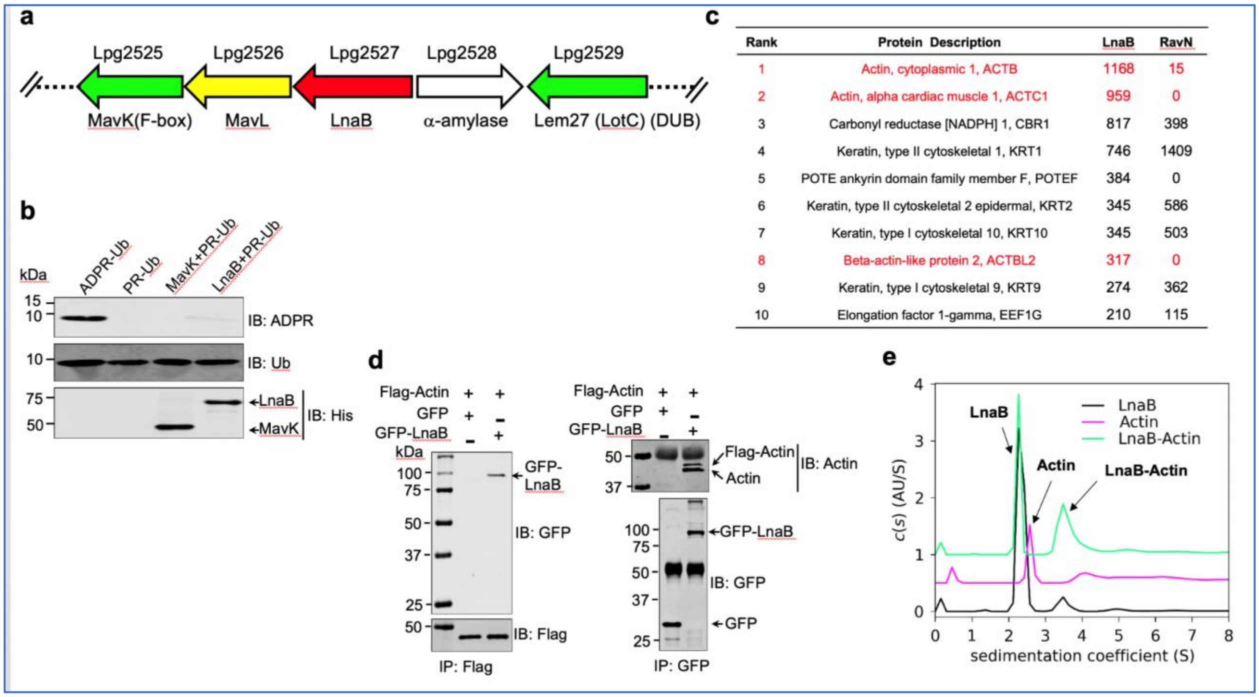

Rank	Protein Description	LnaB	RavN
1	Actin, cytoplasmic 1, ACTB	1168	15
2	Actin, alpha cardiac muscle 1, ACTC1	959	0
3	Carbonyl reductase [NADPH] 1, CBR1	817	398
4	Keratin, type II cytoskeletal 1, KRT1	746	1409
5	POTE ankyrin domain family member F, POTEF	384	0
6	Keratin, type II cytoskeletal 2 epidermal, KRT2	345	586
7	Keratin, type I cytoskeletal 10, KRT10	345	503
8	Beta-actin-like protein 2, ACTBL2	317	0
9	Keratin, type I cytoskeletal 9, KRT9	274	362
10	Elongation factor 1-gamma, EEF1G	210	115

Supplementary Figure 7 Identification of Actin as the co-factor of LnaB

a. The organization of genes in the *mavL* locus. Upstream of *mavL* is *mavK*, which is predicted to have similarity to F-box proteins known to be involved in ubiquitination. *lnaB* and the deubiquitinase *lotC* (also known as *lem27*) is separated by a gene predicted to code for an α-amylase.

b. LnaB or MavK alone cannot convert PR-Ub into ADPR-Ub. Recombinant MavK or LnaB was incubated with PR-Ub for 2 h at 37°C and the production of ADPR-Ub was probed by immunoblotting (upper panel). The presence of ubiquitin, MavK and LnaB in the reactions was detected by immunoblotting with the indicated antibodies (middle and lower panels) (b).

c. Actin was abundantly identified in immunoprecipitation products obtained with beads coated with the Flag antibody from lysates of cells transfected to express Flag-LnaB.

d. Interactions between LnaB and Actin determined by immunoprecipitation. Lysates of 293HEKT cells transfected to express Flag-Actin and GFP-LnaB were subjected to reciprocal immunoprecipitation with beads coated with the Flag antibody or GFP-specific antibodies and protein A. The precipitates resolved by SDS-PAGE were detected by immunoblotting with appropriate antibodies.

e. Binding between Actin and LnaB determined by analytic ultracentrifugation.

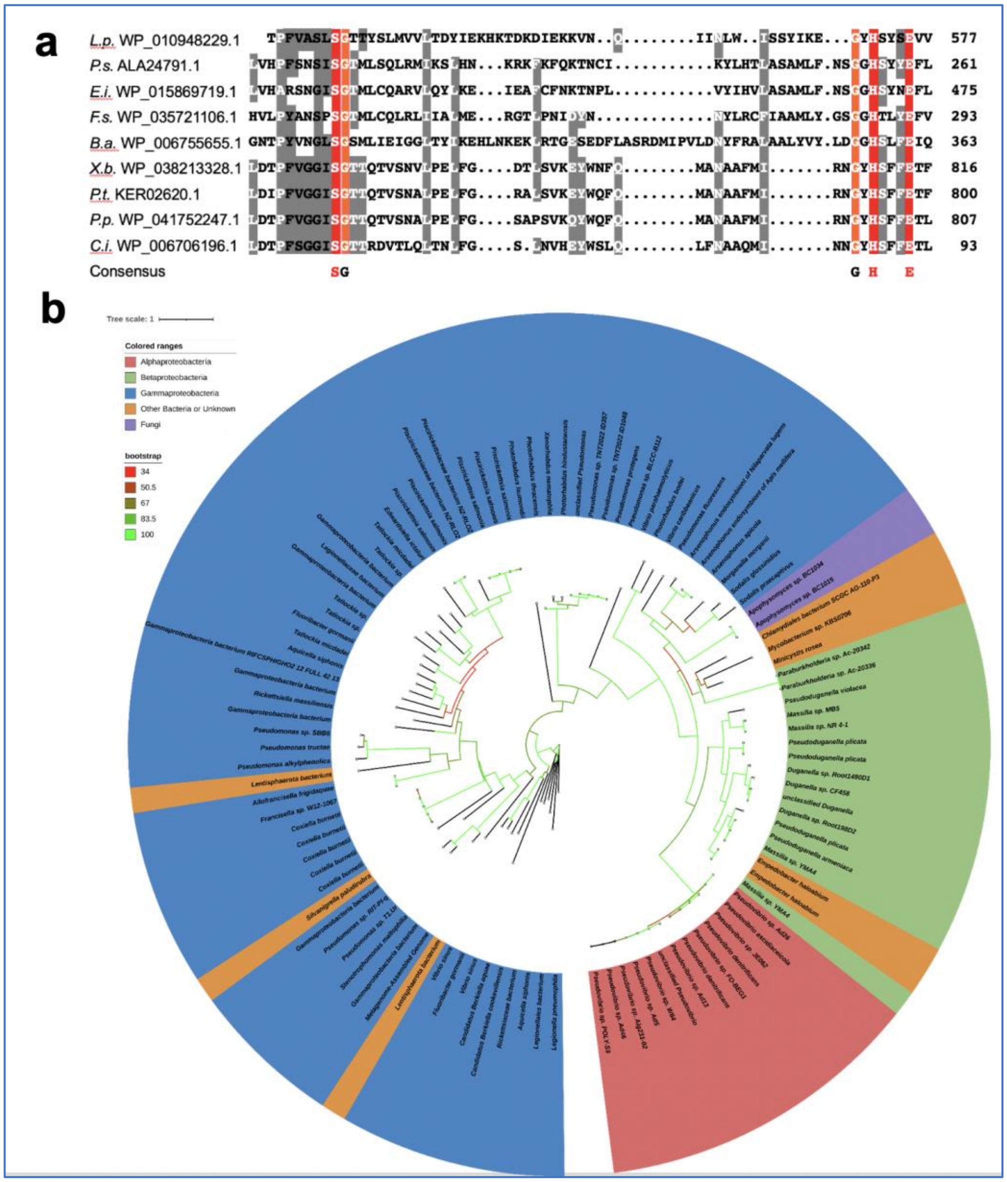

Supplementary Figure 8 LnaB is a member of a family of toxins sharing a conserved S-HxxxE catalytic motif

a. Multiple sequence alignment of the S-HXXXE motifs from diverse bacteria species. The S-HXXXE family was identified by PSI-BLAST, and manually aligned. Listed from left to right are species, accession number, and amino acids. Identities are highlighted in red or orange. Similarities are shaded in gray. The species listed are *Legionella pneumophila* (*L.p.*), *Piscirickettsia salmonis* (*P.s.*), *Edwardsiella ictaluri* (*E.i.*), *Francisella sp.* W12-1067 (*F.s.*), *Burkholderia ambifaria* (*B.a.*), *Xenorhabdus bovienii* (*X.b.*), *Photorhabdus temperate* (*P.t.*), *Pseudomonas protegens* (*P.p.*), *Candidatus Regiella insecticola* (*C.i.*).

b. Phylogenetic tree generated by using the VT+F+R4 subsidence model and 1,000 replicated bootstrap analysis with the IQ-Tree software. The color of each branch represents the taxonomy information of each protein sequence with the annotation to the left of the figure. The bootstrap value is represented by the color of the branches; the minimum value is 60, and it is represented in red by scaling the gradient up to 100, which is colored green.

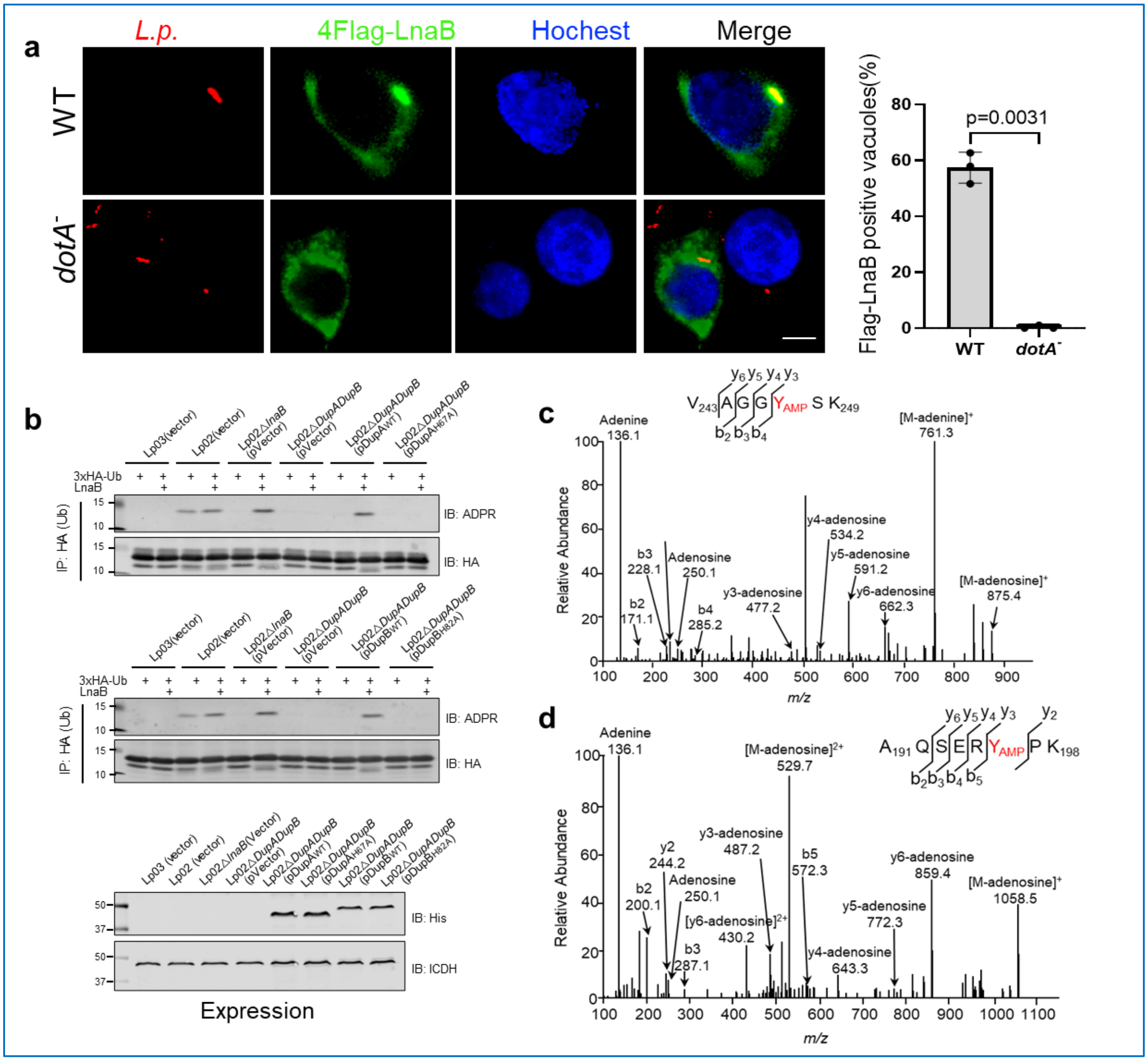

Supplementary Figure 9 PR-Ub in *L. pneumophila*-infected cells was produced by reversal of modification by DupA and DupB

a. Ectopically expressed LnaB is assocated with the *L. pneumophila* vacuole. HEK293 cells transfected to express 4Flag-LnaB were infected with the indicated *L. pneumophila* strains for 2 h and the samples were stained with the appropriate antibodies. Shown are representative images of the bacterial vacuoles of the wild-type and the *dotA* mutant. Quantitation of the association was performed by counting at least 100 vacuoles for each sample. Results shown were from 3 independent experiments each done in triplicate.

b. 293HEK cells transfected to express 3xHA-Ub were infected with opsonized bacteria of the indicated *L. pneumophila* strains and the accumulation of PR-Ub was determined

by adding recombinant LnaB to lysates of infected cells. Note that expression of DupA or DupB but not their catalytically inactive mutants restored the accumulation of PR-Ub in infected cells.

c-d. Identification of self-AMPylation sites on LnaB. The ms/ms spectra of the two tryptic peptides that carried the modified tyrosine residues.

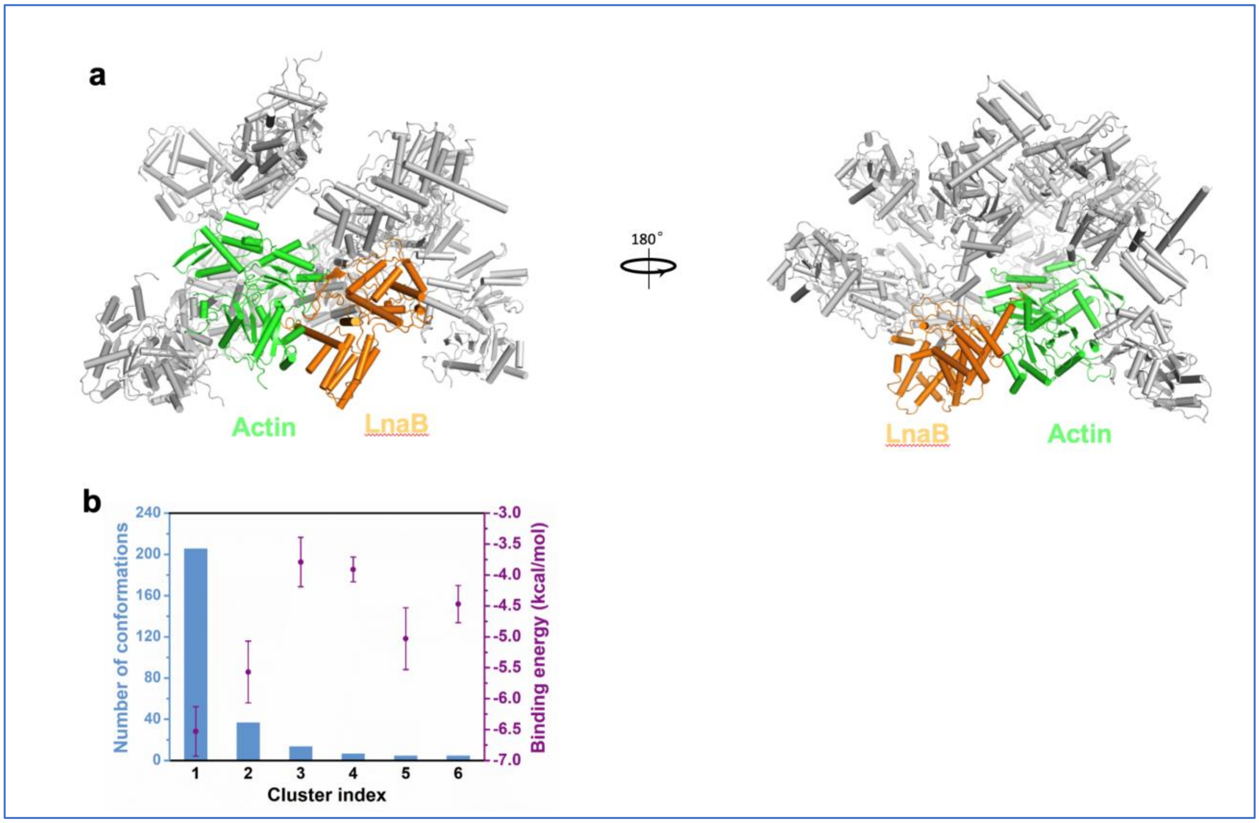

Supplementary Figure 10 Overall structure of the LnaB–actin binary complex in one asymmetric unit (ASU) and a model for the ubiquitin recycling in cells infected by *L. pneumophila*.

a.Two views of the structure of the LnaB-Actin heterodimer in the asymmetric unit displayed as a ribbon diagram; one of the LnaB–Actin heterodimers is colored as shown in Fig. 5 and the other one is colored in grey.

b. Cluster index of the docking of LnaB with ATP. The numbers of conformations and mean binding energy (in kcal/mol) for each cluster are represented by blue columns and purple dots, respectively. All the docked conformations were clustered using the default parameters implemented in AutoDockTools

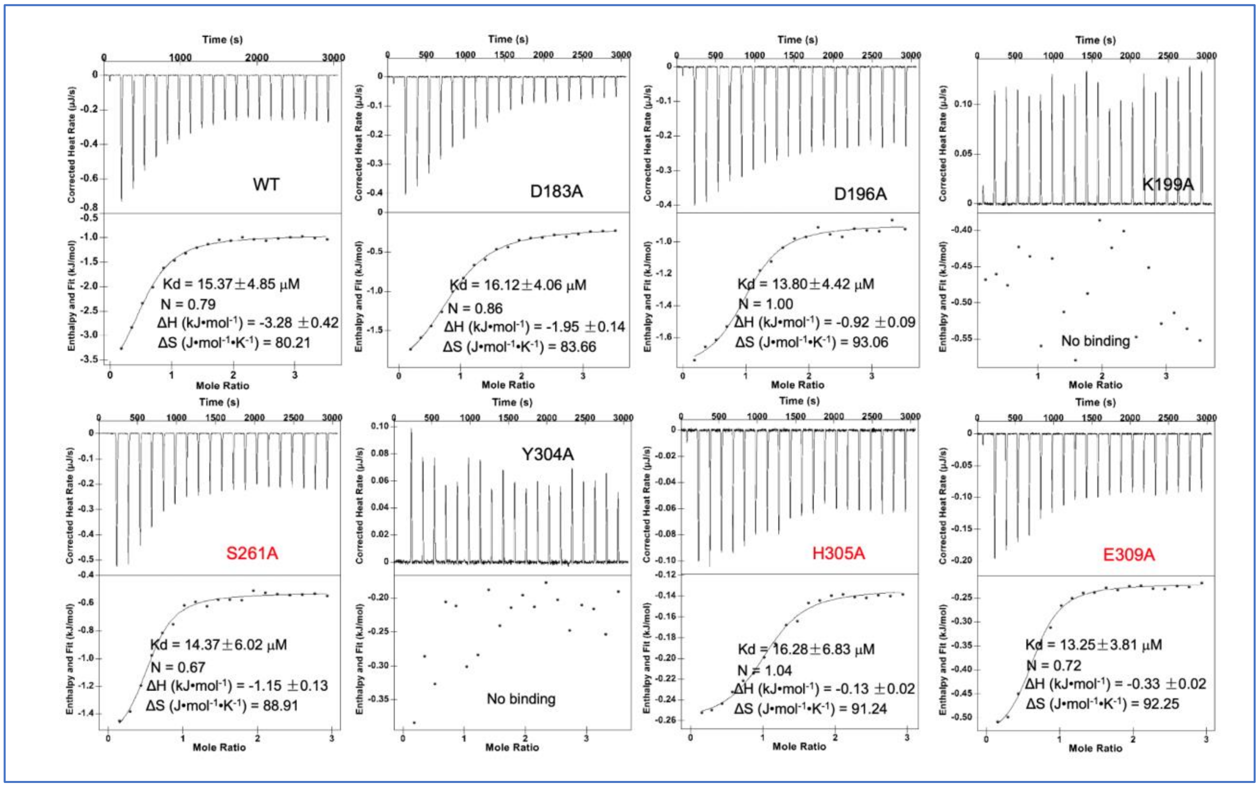

Supplementary Figure 11 Binding affinity between ATP and LnaB and its mutants determined using isothermal titration calorimetry (ITC). These were the original results for Fig. 6i. The binding affinity, stoichiometry and thermodynamic parameters are also shown.

Supplementary Table 1. X-ray crystallography data collection and refinement statistics

Dataset	MavL-ADPR	MavL (D315A)-ADPR-Ub	LnaB-Actin
**Data collection**			
Beamline	BL-17U1	BL-19UF	BL-02U1
Wavelength (Å)	0.979183	0.979183	0.979183
Space group	$P2_12_12_1$	$C121$	$P3_1$
Unit Cell			
a, b, c (Å)	50.18, 107.23, 121.83	80.95, 68.18, 85.55	110.73, 110.73, 406.52
α, β, γ (°)	90.00, 90.00, 90.00	90.00, 100.55, 90.00	90.00, 90.00, 120.00
Resolution (Å)	29.11-2.38 (2.44 -2.38)	41.92-2.00 (2.07-2.00)	39.14-3.61 (3.81-3.61)
Unique reflections	27162 (1970)	30800 (3042)	64013 (9446)
R-merge	0.169 (1.604)	0.323 (0.682)	0.262 (0.826)
Mean I/sigma(I)	11.8 (1.9)	9.7 (2.6)	4.5 (2.4)
$CC_{1/2}$	0.998 (0.609)	0.978 (0.861)	0.965 (0.572)
R-pim	0.048 (0.460)	0.135 (0.299)	0.139 (0.439)
R-meas	0.176 (1.670)	0.351 (0.747)	0.297 (0.937)
Completeness (%)	99.9 (100.0)	99.50 (98.80)	99.8 (100)
Multiplicity	13.1	6.6	4.5
**Refinement**			
Reflections used in refinement	30115 (2903)	30364 (2920)	63970 (6370)
Reflections used for R-free	1998 (192)	1976 (192)	3181 (352)
R-work	0.200(0.307)	0.185(0.270)	0.231(0.202)
R-free	0.245(0.356)	0.222(0.347)	0.275(0.246)
Wilson B-factor (Å^2)	62.84	19.77	73.56
Number of non-hydrogen atoms	5685	3894	30228
Macromolecules	5597	3415	30122
Protein residues	721	435	3791
RMS(bonds)	0.013	0.008	0.005
RMS(angles)	1.66	1.18	1.02
Ramachandran favored (%)	96.78	96.50	92.00
Ramachandran allowed (%)	3.08	3.26	7.00
Ramachandran Outliers (%)	0.14	0.00	0.81
Rotamer outliers (%)	6.50	0.00	3.8
Clashscore	4.85	4.12	11.44
Average B-factor (Å^2)	74.75	20.89	62.75

Statistics for the highest-resolution shell are shown in parentheses.

Supplementary Table 2. Primers used in this study

Primer	Sequence（5'-3'）
LnaB-F	CTGAGATCTTTGTTATATTTTGTACTATGTGA
LnaB-R	CTGCTCGAGCTATTGTAAACGAGCG
LnaB-S261A-F	GTAGGTTGTGCCAGCCAGCGACGCCACAA
LnaB-S261A-R	TTGTGGCGTCGCTGGCTGGCACAACCTAC
LnaB-H305A-F	ACCTCACTGTAGCTGGCATAACCTTCCTTGATATAACTGGAAATC
LnaB-H305A-R	GATTTCCAGTTATATCAAGGAAGGTTATGCCAGCTACAGTGAGGT
LnaB-E309A-F	GTCAAAACATCCACAACCGCACTGTAGCTGTGATAAC
LnaB-E309A-R	GTTATCACAGCTACAGTGCGGTTGTGGATGTTTTGAC
LnaB-Y196F-F	ATCTGTCCTTTTTTGGGAATCTCTCTGATTGTGCTTTTAAAGG
LnaB-Y196F-R	CCTTTAAAAGCACAATCAGAGAGATTCCCAAAAAAGGACAGAT
LnaB-Y247F-F	GTAGGCTTAGTTTTTGAAAACCCACCCGCCACTTTC
LnaB-Y247F-R	GAAAGTGGCGGGTGGGTTTTCAAAAACTAAGCCTAC
LnaB D183A-F	TCTTTTGTCATGCTGAATGCAAATGTTGAATTACCTTTA
LnaB D183A-R	TAAAGGTAATTCAACATTTGCATTCAGCATGACAAAAGA
LnaB Y196A-F	AAAGCACAATCAGAGAGAGCACCAAAAAAGGACAGATCC
LnaB Y196A-R	GGATCTGTCCTTTTTTGGTGCTCTCTCTGATTGTGCTTT
LnaB K199A-F	TCAGAGAGATACCCAAAAGCAGACAGATCCAGAGAAGAG
LnaB K199A-R	CTCTTCTCTGGATCTGTCTGCTTTTGGGTATCTCTCTGA
LnaB R203A-F	CCAAAAAAGGACAGATCCGCAGAAGAGTTGGGGAATACTC
LnaB R203A-R	GAGTATTCCCCAACTCTTCTGCGGATCTGTCCTTTTTTGG
LnaB R232A-F	GATAACCTGGTGCCTGTCGCAGATGTGCCCAAAATAGCC
LnaB R232A-R	GGCTATTTTGGGCACATCTGCGACAGGCACCAGGTTATC
LnaB S261A-F	CCTTTTGTGGCGTCGCTGGCAGGCACAACCTACTCACTT
LnaB S261A-R	AAGTGAGTAGGTTGTGCCTGCCAGCGACGCCACAAAAGG
LnaB Y299A-F	AATTTATGGATTTCCAGTGCAATCAAGGAAGGTTATCAC
LnaB Y299A-R	GTGATAACCTTCCTTGATTGCACTGGAAATCCATAAATT
LnaB Y304A-F	AGTTATATCAAGGAAGGTGCACACAGCTACAGTGAGGTTG
LnaB Y304A-R	CAACCTCACTGTAGCTGTGTGCACCTTCCTTGATATAACT

LnaB H305A-F	TATATCAAGGAAGGTTATGCAAGCTACAGTGAGGTTGTG
LnaB H305A-R	CACAACCTCACTGTAGCTTGCATAACCTTCCTTGATATA
LnaB E309A-F	GGTTATCACAGCTACAGTGCAGTTGTGGATGTTTTGACAG
LnaB E309A-R	CTGTCAAAACATCCACAACTGCACTGTAGCTGTGATAACC
LnaB T209A-F	AGAGAAGAGTTGGGGAATGCACCATCTGCGAATCCAGGG
LnaB T209A-R	CCCTGGATTCGCAGATGGTGCATTCCCCAACTCTTCTCT
LnaB N220A-F	CCAGGGATTATGAAACCTGCATCACCTAATTTTACCGAT
LnaB N220A-R	ATCGGTAAAATTAGGTGATGCAGGTTTCATAATCCCTGG
LnaB T225A-F	CCTAACTCACCTAATTTTGCAGATAACCTGGTGCCTGTC
LnaB T225A-R	GACAGGCACCAGGTTATCTGCAAAATTAGGTGAGTTAGG
LnaB D347A-F	GAATTTCGTAAAGCTCAGGCATATGTTTTTGGTTTGACA
LnaB D347A-R	TGTCAAACCAAAAACATATGCCTGAGCTTTACGAAATTC
LnaB L352A-F	CAGGATTATGTTTTTGGTGCAACAATTCAATCAGCGATG
LnaB L352A-R	CATCGCTGATTGAATTGTTGCACCAAAAACATAATCCTG
LnaB Q355A-F	GTTTTTGGTTTGACAATTGCATCAGCGATGCATCATGAG
LnaB Q355A-R	CTCATGATGCATCGCTGATGCAATTGTCAAACCAAAAAC
LnaB H359A-F	ACAATTCAATCAGCGATGGCACATGAGTTACAAGAGCGA
LnaB H359A-R	TCGCTCTTGTAACTCATGTGCCATCGCTGATTGAATTGT
LnaB E361A-F	CAATCAGCGATGCATCATGCATTACAAGAGCGATTTAAG
LnaB E361A-R	CTTAAATCGCTCTTGTAATGCATGATGCATCGCTGATTG
LnaB L362A-F	TCAGCGATGCATCATGAGGCACAAGAGCGATTTAAGAAT
LnaB L362A-R	ATTCTTAAATCGCTCTTGTGCCTCATGATGCATCGCTGA
LnaB R365A-F	CATCATGAGTTACAAGAGGCATTTAAGAATAAAGAAAAAC
LnaB R365A-R	GTTTTTCTTTATTCTTAAATGCCTCTTGTAACTCATGATG
LnaB K301A-F	TGGATTTCCAGTTATATCGCAGAAGGTTATCACAGCTAC
LnaB K301A-R	GTAGCTGTGATAACCTTCTGCGATATAACTGGAAATCCA
LnaB E302A-F	ATTTCCAGTTATATCAAGGCAGGTTATCACAGCTACAGTG
LnaB E302A-R	CACTGTAGCTGTGATAACCTGCCTTGATATAACTGGAAAT
LnaB 1-558-F	CGGGATCCATGTTATATTTTGTACTATGTG
LnaB 1-558-R	CCCAAGCTTTTATTGTAAACGAGCGCTTT
LnaB 19-441-F	CGGGATCCATGTCATACCAAAAAATAGAAC
LnaB 19-441-R	CCCAAGCTTTTAACCACGATTACTTTTTTGT
LnaB 1-361-F	CGGGATCCATGTTATATTTTGTACTATGTG
LnaB 1-361-R	CCCAAGCTTTTACTCATGATGCATCGCTGAT
Hs-NOD1-sgRNA1-F	CACCGGCAACTCGCAGATGCCTACG

Hs-NOD1-sgRNA1-R	AAACCGTAGGCATCTGCGAGTTGCC
Hs-NOD1-sgRNA2-F	CACCGGCTCACTCAGAGCAAAGTCG
Hs-NOD1-sgRNA2-R	AAACCGACTTTGCTCTGAGTGAGCC
Hs-NOD2-sgRNA1-F	CACCGGGCGCCTTCTGGACACCGTC
Hs-NOD2-sgRNA1-R	AAACGACGGTGTCCAGAAGGCGCCC
Hs-NOD2-sgRNA2-F	CACCGGACGGTGTCCAGAAGGCGCC
Hs-NOD2-sgRNA2-R	AAACGGCGCCTTCTGGACACCGTCC
0750-F	*CGCGGATCC*ATGTCATTAGAATTTTATACAG
0750-R	*ACGCGTCGAC*TTACTTGCCCAT
1483-F	*CGCGGATCC*ATGAGGATAAGACCCCAA
1483-R	*ACGCGTCGAC*TTACACGGTTTTGTCGT
WP0158-F	*CGCGGATCC*ATGAGGGATACTTCAGTAAATACATATG
WP0158-R	*ACGCGTCGAC*TTAGCCATACACGCGC
WP006755-F	*CGCGGATCC*ATGACATATGTAACTGATCACGC
WP006755-R	*ACGCGTCGAC*TTAACGGTGAGTCAGCGG
MAZ443-F	*CGCGGATCC*ATGTCAAAATTGTTAAAAGTACTAAAG
MAZ443-R	*ACGCGTCGAC*TTAGCGTTTGGACTTTTTG
0750-S264A-F	gggagcacgtagtaccggcgagagattccacaaacg
0750-S264A-R	cgtttgtggaatctctcgccggtactacgtgctccc
1483-S513A-F	agccgctaccggcaatagaattaacaaacggcacat
1483-S513A-R	atgtgccgtttgttaattctattgccggtagcggct
WP0158-S448A-F	gcatggtgccggcaataccattgctacgcgcatg
WP0158-S448A-R	catgcgcgtagcaatggtattgccggcaccatgc
WP006755-S333A-F	ttcaatcagcatggaaccggcgagaccattaacatacggc
WP006755-S333A-R	gccgtatgttaatggtctcgccggttccatgctgattgaa
MAZ443-S245A-F	cgtacgtagtaccggcaatggaattaacaaacggagcttgaga
MAZ443-S245A-R	tctcaagctccgtttgttaattccattgccggtactacgtacg
MavL-FL-F	CCGGAATTCTTGAGATTGTTTCAGTACAATCAAC
MavL-FL-R	CGGGGTACCTTACTGAGGACCCGATTTTTTCGAT
MavL (40-404)-F	CGCGGATCCATGATAATGGCCTATCAATTGTTGC
MavL (40-404)-R	CCGCTCGAGTTATTCCAAGGTATCCAATTTACCG
Ub-F	CGCGGATCCATGCAGATCTTCGTGAAAACCCTGA
Ub-R	CCGCTCGAGTTAACCACCTCTCAGACGCAGGACC
MavL-D315A-F	ATTGTTGCCTGGGCTCATTTTTCCTGGCCCG
MavL-D315A-R	TGAAACGAGGATATGCGTATCTGGATTGCT

MavL-N322A-F	TCCTGGCCCGGAGCTGATTATTGGGGCGGT
MavL-N322A-R	AAAATGATCCCAGGCAACAATTGAAACGAGGATAT
MavL-D323A-F	TCCTGGCCCGGAAATGCTTATTGGGGCGGT
MavL-D323A-R	AAAATGATCCCAGGCAACAATTGAAACGAG
MavL-D333A-F	CGTCAAACAGATGCTGGTGTCAAGGCCGCT
MavL-D333A-R	AGCACCGCCCCAATAATCATTTCCGGGCCA
MavL-K236A-F	TATGATGTGATTGCGCCCTATGTCAGA
MavL-K236A-R	ATAAGCGCCTGAAAAACACCCTGTTCCT
