# Supplementary material for: Legionella maintains host cell ubiquitin homeostasis by effectors with unique catalytic mechanisms
Source: Nat Commun. 2024 Jul 15;15:5953. doi: 10.1038/s41467-024-50311-2 (PMC11251166; doi:10.1038/s41467-024-50311-2)
Supplement: Supplementary file 3 — Description of Additional Supplementary Files [file 41467_2024_50311_MOESM3_ESM.pdf]

Description of Additional Supplementary Files:

Supplementary Data 1: LnaB interacting proteins identified by IP-MS
