# Supplementary material for: Legionella maintains host cell ubiquitin homeostasis by effectors with unique catalytic mechanisms
Source: Nat Commun. 2024 Jul 15;15:5953. doi: 10.1038/s41467-024-50311-2 (PMC11251166; doi:10.1038/s41467-024-50311-2)
Supplement: Supplementary file 5 — Reporting Summary [file 41467_2024_50311_MOESM5_ESM.pdf]

Corresponding author(s): Zhao-qing Luo
Last updated by author(s): Jun 19, 2024

Reporting Summary

Nature Portfolio wishes to improve the reproducibility of the work that we publish. This form provides structure for consistency and transparency in reporting. For further information on Nature Portfolio policies, see our Editorial Policies and the Editorial Policy Checklist.

Statistics

For all statistical analyses, confirm that the following items are present in the figure legend, table legend, main text, or Methods section.

n/a	Confirmed	
☐	☒	The exact sample size (*n*) for each experimental group/condition, given as a discrete number and unit of measurement
☐	☒	A statement on whether measurements were taken from distinct samples or whether the same sample was measured repeatedly
☐	☒	The statistical test(s) used AND whether they are one- or two-sided<br>*Only common tests should be described solely by name; describe more complex techniques in the Methods section.*
☒	☐	A description of all covariates tested
☐	☒	A description of any assumptions or corrections, such as tests of normality and adjustment for multiple comparisons
☐	☒	A full description of the statistical parameters including central tendency (e.g. means) or other basic estimates (e.g. regression coefficient) AND variation (e.g. standard deviation) or associated estimates of uncertainty (e.g. confidence intervals)
☐	☒	For null hypothesis testing, the test statistic (e.g. *F*, *t*, *r*) with confidence intervals, effect sizes, degrees of freedom and *P* value noted<br>*Give P values as exact values whenever suitable.*
☒	☐	For Bayesian analysis, information on the choice of priors and Markov chain Monte Carlo settings
☒	☐	For hierarchical and complex designs, identification of the appropriate level for tests and full reporting of outcomes
☒	☐	Estimates of effect sizes (e.g. Cohen's *d*, Pearson's *r*), indicating how they were calculated

Our web collection on statistics for biologists contains articles on many of the points above.

Software and code

Policy information about availability of computer code

Data collection	Odyssey Licor imaging system, Q Exactive™ HF Hybrid Quadrupole-Orbitrap Mass Spectrometer (Thermo Scientific)
Data analysis	Graphpad Prism 8.0.1, PyMOL 2.5, MaxQuant 1.6.3.3, Xcalibur Qual Browser 2.0, PSI-BLAST, COOT 0.8.9.1, PHENIX 1.17.1-3660. Supplementary Figure 1a was created by Microsoft Powerpoint 2010.

For manuscripts utilizing custom algorithms or software that are central to the research but not yet described in published literature, software must be made available to editors and reviewers. We strongly encourage code deposition in a community repository (e.g. GitHub). See the Nature Portfolio guidelines for submitting code & software for further information.

Data

Policy information about availability of data

All manuscripts must include a data availability statement. This statement should provide the following information, where applicable:

- Accession codes, unique identifiers, or web links for publicly available datasets
- A description of any restrictions on data availability
- For clinical datasets or third party data, please ensure that the statement adheres to our policy

Structure factors and atomic coordinates has been deposited in the Protein Data Bank (PDB) under accessions: MavL-ADPR (PDB:8IPW), MavL40-404(D315A)-ADPR-Ub (PDB:8IPJ), LnaB-actin (PDB:8J9B). The data that support the conclusions of this study are included in this published article along with its Supplementary

Information files, and are also available from the corresponding author upon request. LnaB interacting proteins identified by IP-MS are included in Supplementary Dataset 1, including full gels and blots are provided in the Source Data file. Source data are provided as a Source Data file.

Research involving human participants, their data, or biological material

Policy information about studies with human participants or human data. See also policy information about sex, gender (identity/presentation), and sexual orientation and race, ethnicity and racism.

Reporting on sex and gender	No human research participant in this study
Reporting on race, ethnicity, or other socially relevant groupings	No human research participant in this study
Population characteristics	No human research participant in this study
Recruitment	No human research participant in this study
Ethics oversight	No human research participant in this study

Note that full information on the approval of the study protocol must also be provided in the manuscript.

Field-specific reporting

Please select the one below that is the best fit for your research. If you are not sure, read the appropriate sections before making your selection.

☒ Life sciences ☐ Behavioural & social sciences ☐ Ecological, evolutionary & environmental sciences

For a reference copy of the document with all sections, see nature.com/documents/nr-reporting-summary-flat.pdf

Life sciences study design

All studies must disclose on these points even when the disclosure is negative.

Sample size	No sample size calculation was performed
Data exclusions	No data generated was excluded from initial analysis
Replication	All data replicated for 3 times, all attempts at replication were successful.
Randomization	Not applicable in this study
Blinding	Not applicable in this study

Reporting for specific materials, systems and methods

We require information from authors about some types of materials, experimental systems and methods used in many studies. Here, indicate whether each material, system or method listed is relevant to your study. If you are not sure if a list item applies to your research, read the appropriate section before selecting a response.

Materials & experimental systems

n/a	Involved in the study	
☐	☒	Antibodies
☐	☒	Eukaryotic cell lines
☒	☐	Palaeontology and archaeology
☒	☐	Animals and other organisms
☒	☐	Clinical data
☒	☐	Dual use research of concern
☒	☐	Plants

Methods

n/a	Involved in the study	
☒	☐	ChIP-seq
☒	☐	Flow cytometry
☒	☐	MRI-based neuroimaging

Antibodies

Antibodies used	Purified His6-GFP and His6-GST proteins were used to raise rabbit specific antibodies using a standard protocol (Pocono Rabbit Farm & Laboratory). These antibodies were affinity purified as described. Antibodies specific for SdeA had been described (PMID:

	27049943). For immunoblotting, samples resolved by SDS-PAGE were transferred onto 0.2 µm nitrocellulose membranes (Bio-Rad, cat# 1620112), which were blocked with 5% non-fat milk or 3% BSA at R.T. for 1 h prior to being incubated with the appropriate primary antibodies: anti-Flag (Sigma, cat# F1804), 1:5000; anti-Actin (MP Biochemicals, cat# 0869100), 1:5000; anti-tubulin (DSHB, E7) 1:10,000; anti-ADPR (Sigma, cat# MABE1016), 1:1000, anti-His (Sigma, cat# H1029), 1:5000; anti-Ub (Santa Cruz, P4D1, cat# sc-8017), 1:1000.
Validation	1. anti-Flag (Sigma, cat# F1804): Application statement in manufacturer's website as follows: https://www.sigmaaldrich.cn/CN/zh/product/sigma/f1804<br><br>2. anti-Actin (MP Biochemicals, cat# 0869100): Application statement in manufacturer's website as follows: https://www.mpbiochina.com/cn/anti-actin-mouse-monoclonal-antibody-clone-c4<br><br>3. anti-tubulin (DSHB, E7): Application statement in manufacturer's website as follows: https://dshb.biology.uiowa.edu/E7_2<br><br>4. anti-ADPR (Sigma, cat# MABE1016): Application statement in manufacturer's website as follows: https://www.sigmaaldrich.cn/CN/zh/product/mm/mabe1016<br><br>5. anti-His (Sigma, cat# H1029): Application statement in manufacturer's website as follows: https://www.sigmaaldrich.cn/CN/zh/product/sigma/h1029<br><br>6. anti-Ub (Santa Cruz, P4D1, cat# sc-8017): Application statement in manufacturer's website as follows: https://www.scbt.com/zh/p/ubiquitin-antibody-p4d1

Eukaryotic cell lines

Policy information about cell lines and Sex and Gender in Research

Cell line source(s)	293T cell line (Cat No. CRL-3216) were obtained from American Type Culture Collection (ATCC).
Authentication	Authenticated by ATCC
Mycoplasma contamination	Contamination tested by using the universal mycoplasma detection kit from ATCC (cat# 30-1012K).
Commonly misidentified lines (See ICLAC register)	No commonly misidentified lines were used in this study.

Plants

Seed stocks	No plants were used in this study.
Novel plant genotypes	No plants were used in this study.
Authentication	No plants were used in this study.
