# Supplementary material for: Legionella maintains host cell ubiquitin homeostasis by effectors with unique catalytic mechanisms
Source: Nat Commun. 2024 Jul 15;15:5953. doi: 10.1038/s41467-024-50311-2 (PMC11251166; doi:10.1038/s41467-024-50311-2)

Fig.1a Native PAGE CBB Stain

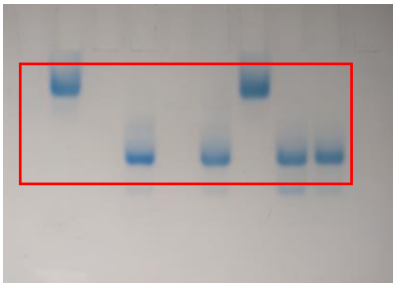

Fig.1a SDS-PAGE CBB Stain

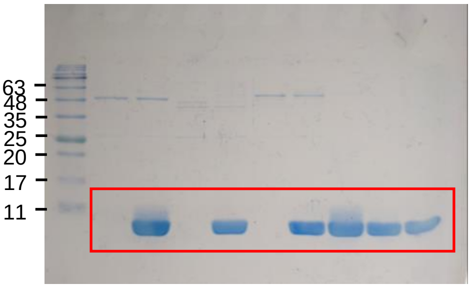

Fig.1a Phosphoprotein stain

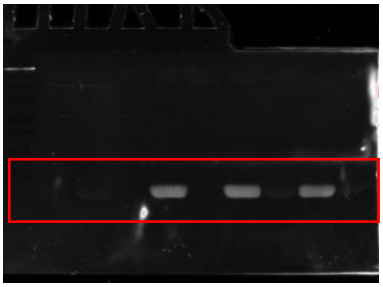

Fig.1a IB:ADPR

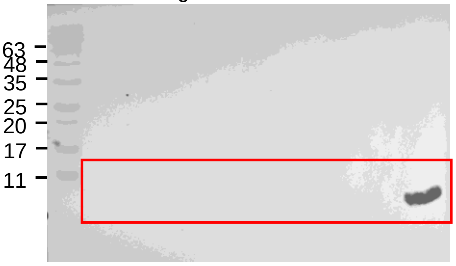

Fig.1b IB:ADPR

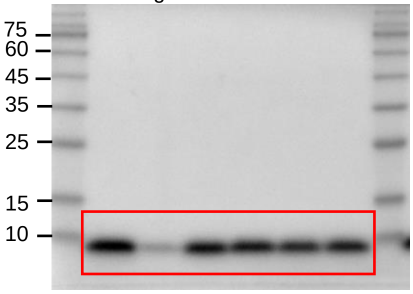

Fig.1b IB:Ub

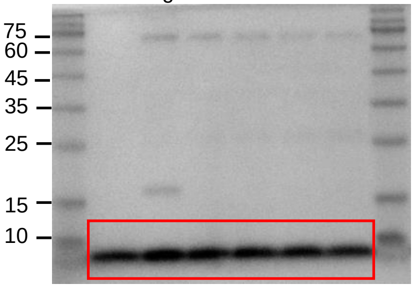

Fig.1b MavL (CBB Stain)

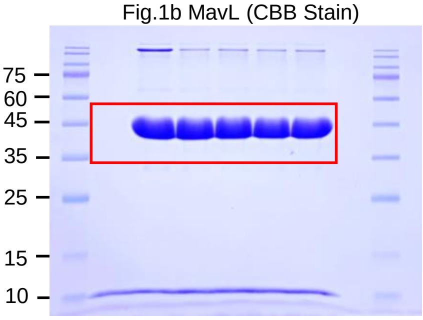

Fig.1f IB:ADPR

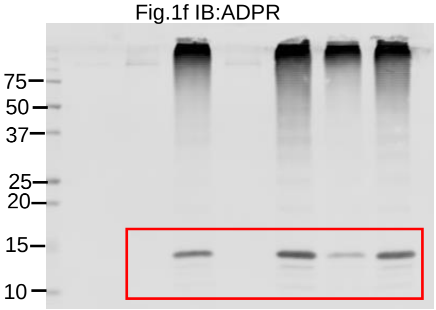

Fig.1f IB:HA(Ub)

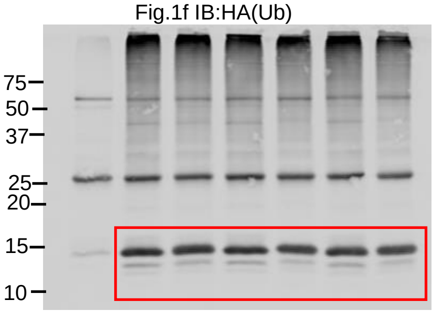

Fig.1f IB:Flag

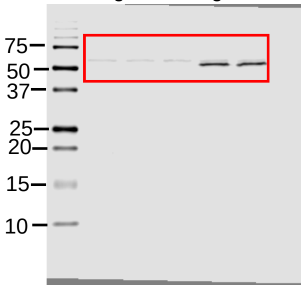

Fig.1f IB:ICDH

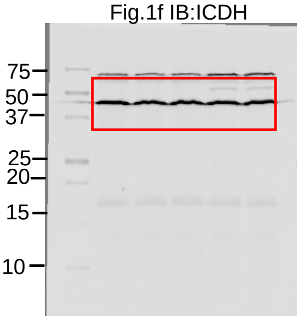

Fig.2a IB:ADPR

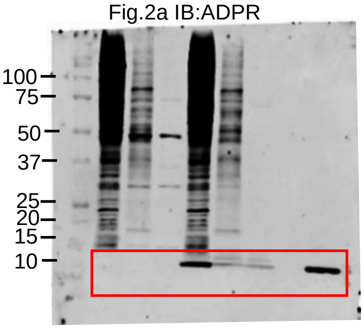

Fig.2a IB:Ub

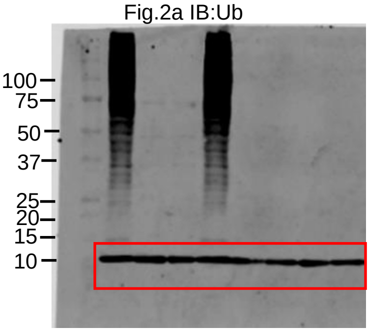

Fig.2a IB:His

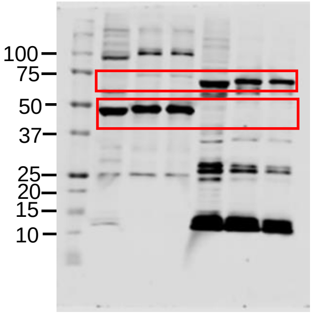

Fig.2d IB:ADPR

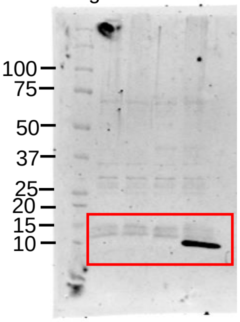

Fig.2d IB:Ub

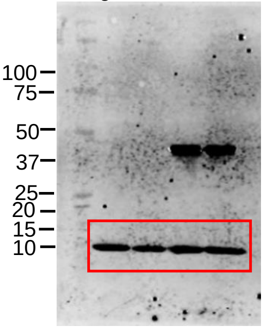

Fig.2d IB:His

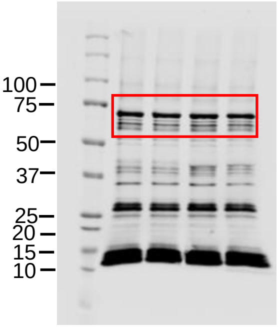

Fig.2d IB:actin

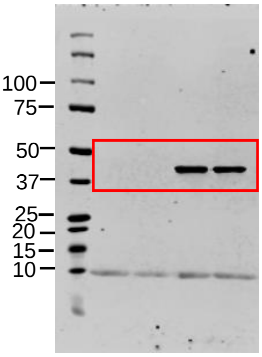

Fig.2f

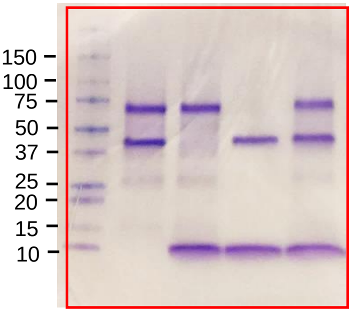

Fig.2f

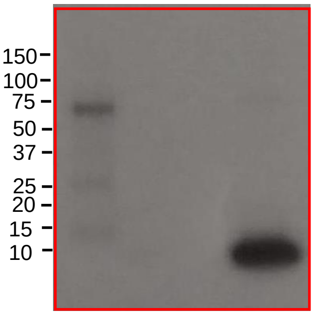

Fig.2f IB:ADPR

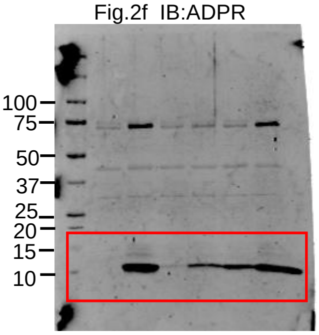

Fig.2f IB:Ub

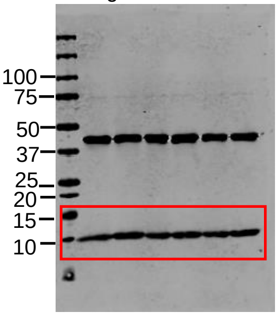

Fig.2f IB:His

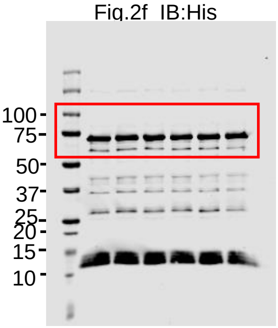

Fig.2f IB:actin

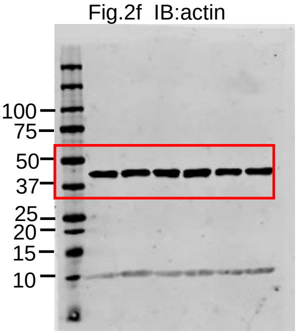

Extended Fig.3d IB:Flag

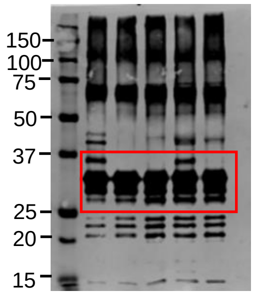

Extended Fig.3d IB:His and actin

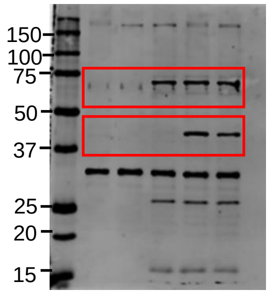

Extended Fig.3d IB:Ub

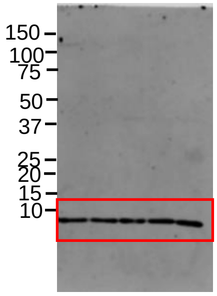

Extended Fig.3d IB:SdeA

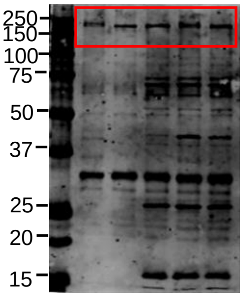

Extended Fig.3e IB:Ub

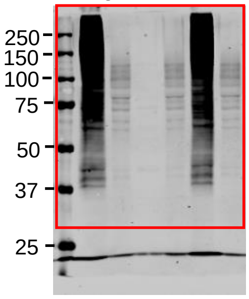

Extended Fig.3d IB:His and actin

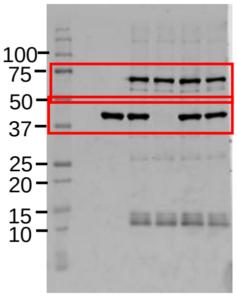

Extended Fig.3d IB:GST

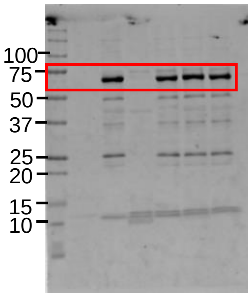

Fig.4a IB:ADPR

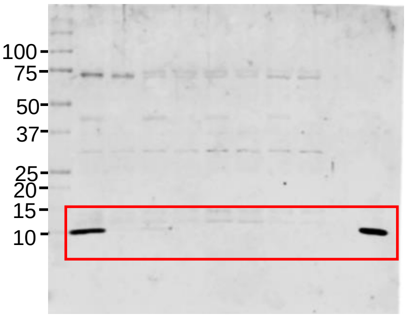

Fig.4a IB:Ub

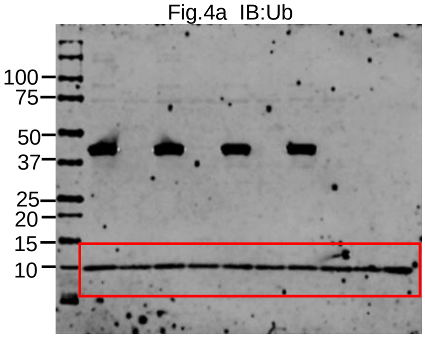

Fig.4a IB:His

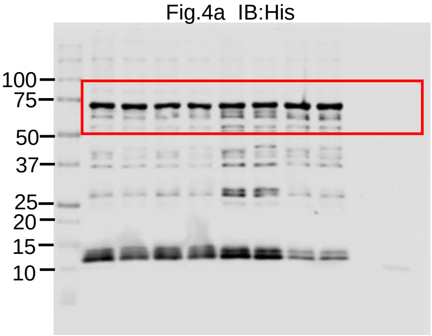

Fig.4a IB:actin

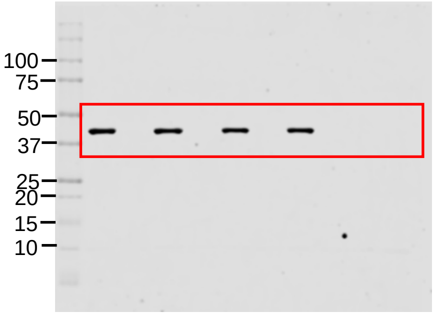

Fig.4b

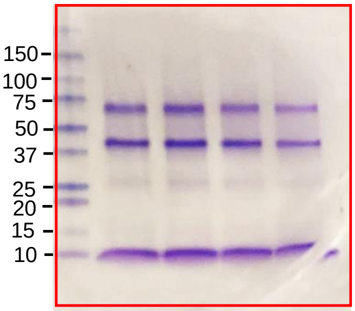

Fig.4b

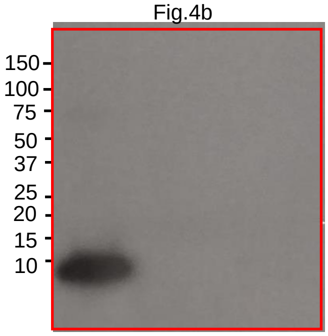

Fig.4d IB:ADPR

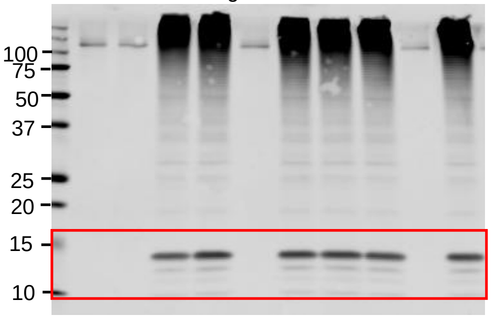

Fig.4d IB:HA

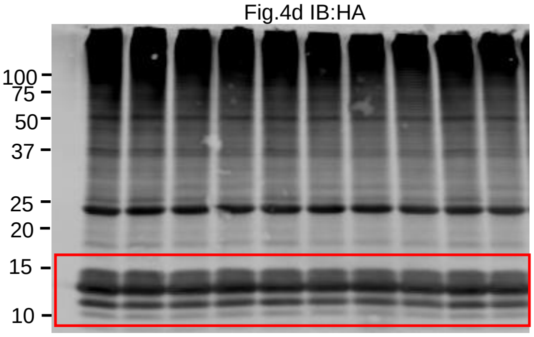

Fig.4d IB:His

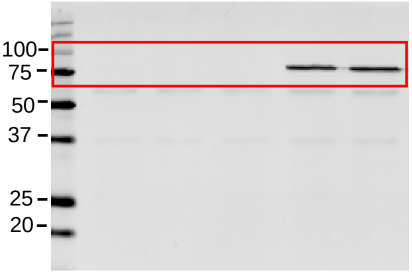

Fig.4d IB:ICDH

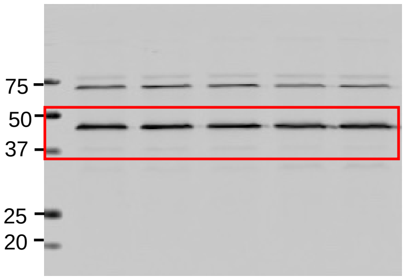

Fig.4f IB:SdeA (Left)

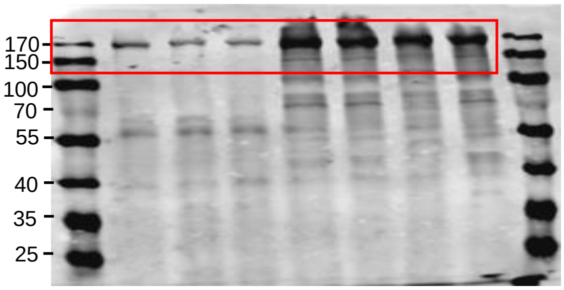

Fig.4f IB:ICDH (Left)

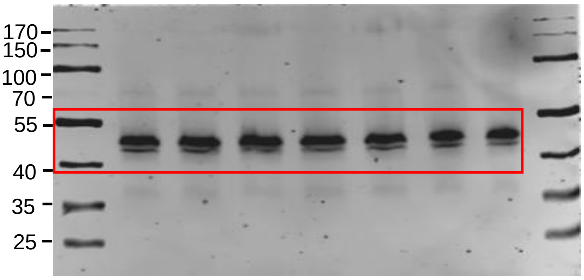

Fig.4f IB:SdeA (right)

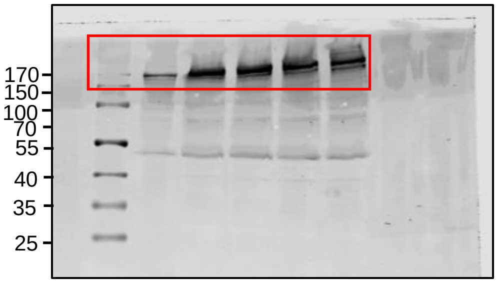

Fig.4f IB: Flag-LnaB (right)

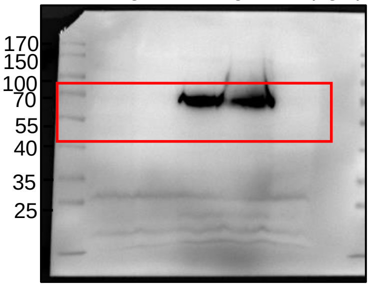

Fig.4f IB: ICDH (right)

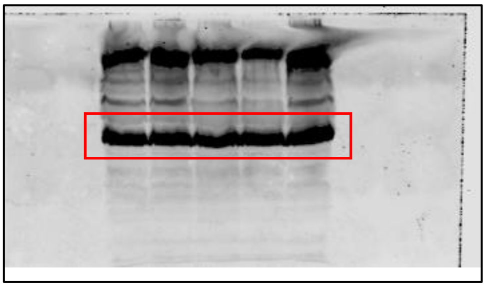

Fig.5b

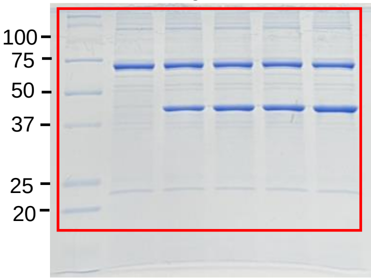

Fig.5b

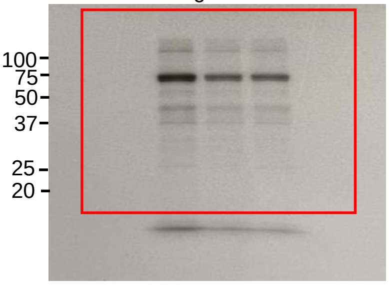

Fig.5c IB:ADPR

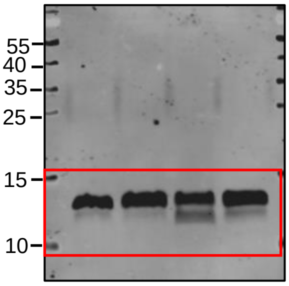

Fig.5c

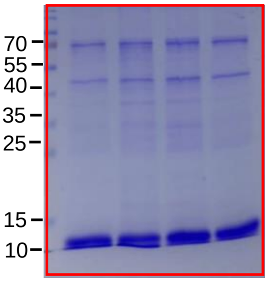

Fig.5d

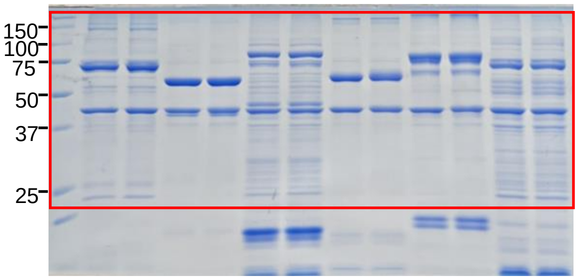

Fig.5d

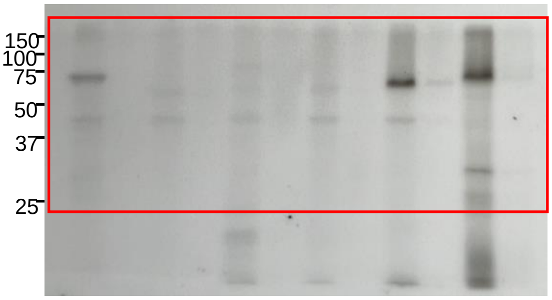

Fig.5e IB:Flag

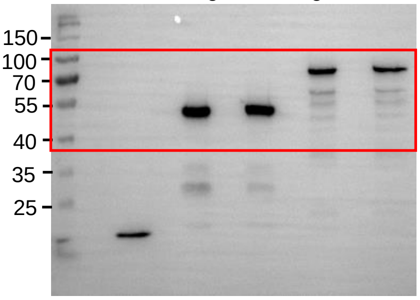

Fig.5e IB:PGK

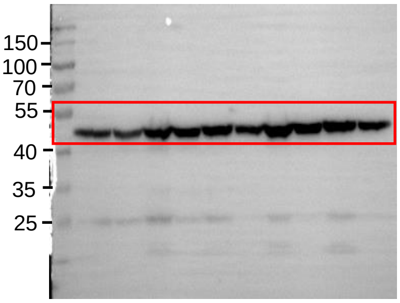

Fig.5e IB:Flag

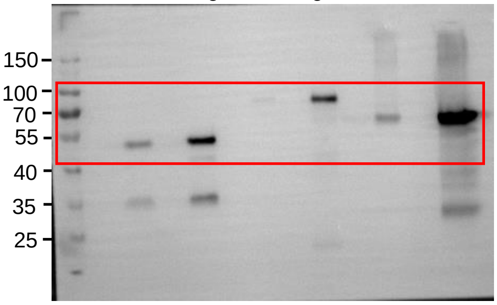

Fig.5e IB:PGK

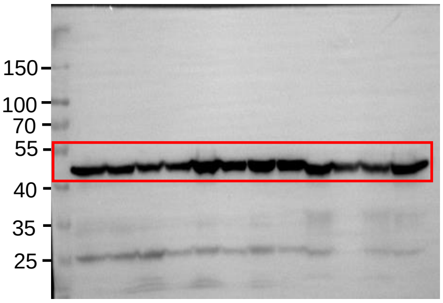

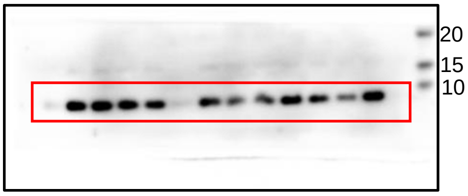

Fig.6e IB:ADPR

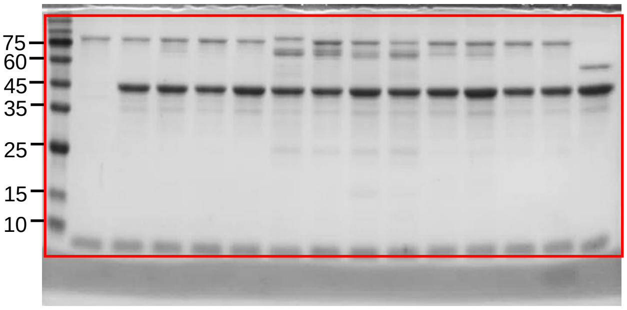

Fig.6e CBB stain

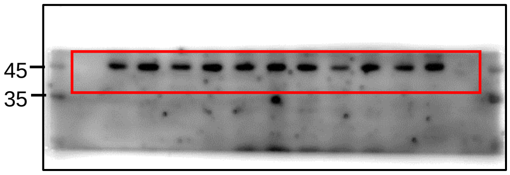

Fig.6f IB:actin

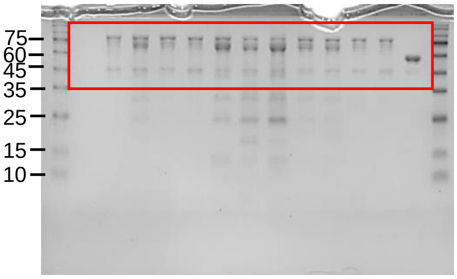

Fig.6f IP CBB stain

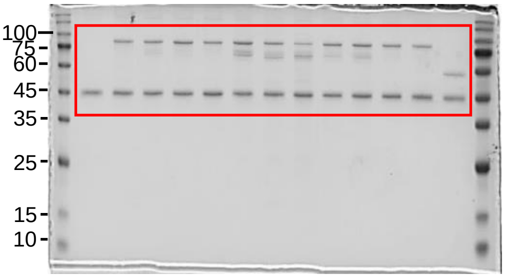

Fig.6f Input CBB stain

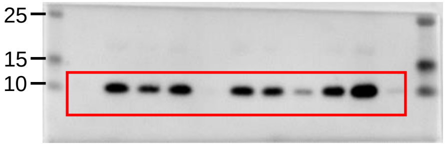

Fig.6h IB:ADPR

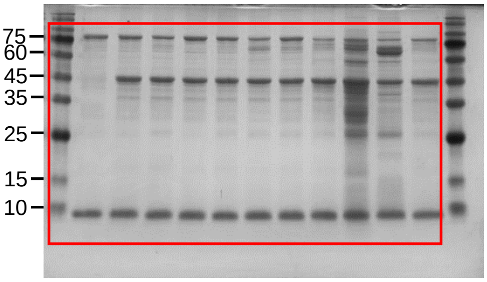

Fig.6h CBB stain

Extended Fig.1c IB:Ub

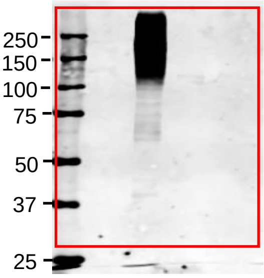

Extended Fig.2d IB:ADPR

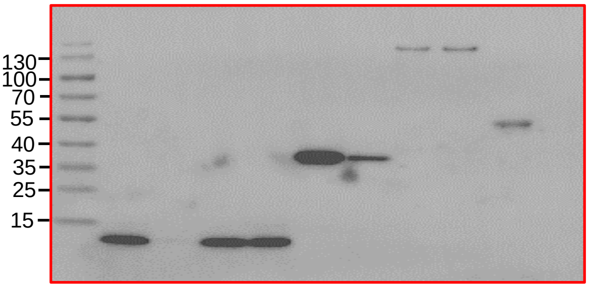

Extended Fig.2d CBB Stain

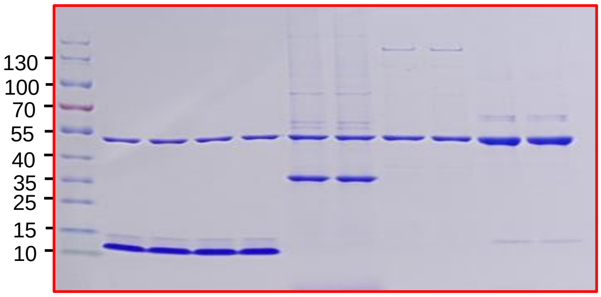

Extended Fig.2e native PAGE

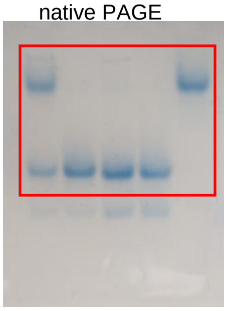

Extended Fig.2e IB:GST

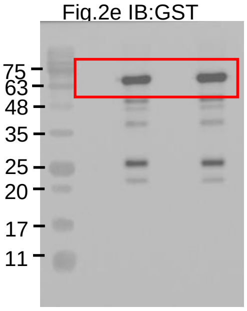

Extended Fig.2e IB:Ub

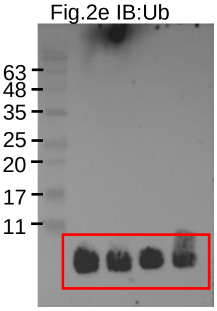

Extended Fig.2e IB:ADPR

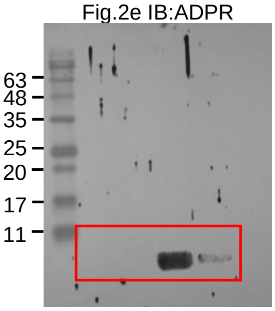

Extended Fig.7b IB:ADPR

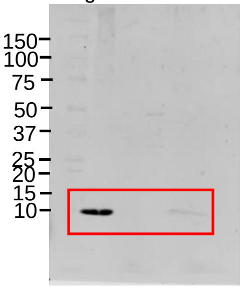

Extended Fig.7b IB:Ub

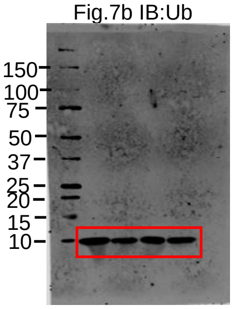

Extended Fig.7b IB:His

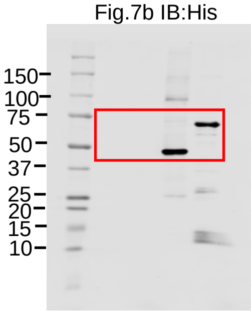

Extended Fig.7d IP:Flag IB:GFP

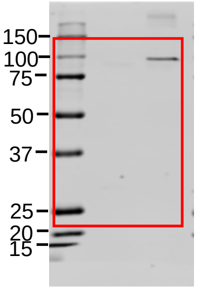

Extended Fig.7d IP:Flag IB:Flag

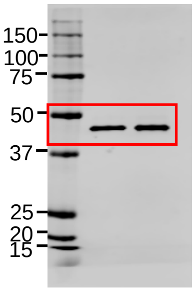

Extended Fig.7d IP:GFP IB:actin

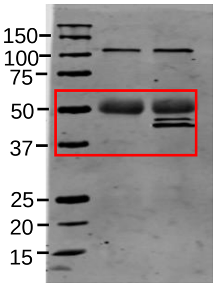

Extended Fig.7d IP:GFP IB:GFP

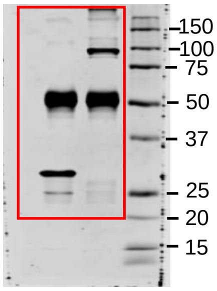

Extended Fig.9b
IB:ADPR (upper panel)

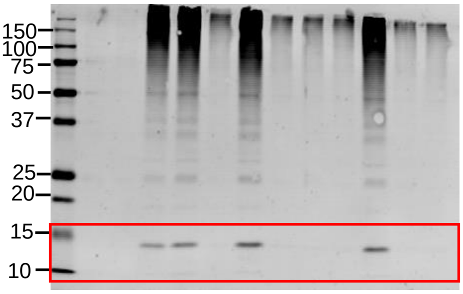

Extended Fig.9b
IB:HA (upper panel)

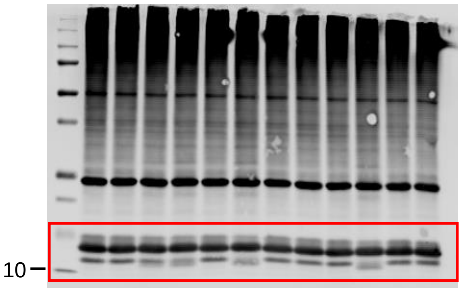

Extended Fig.9b
IB:ADPR (middle panel)

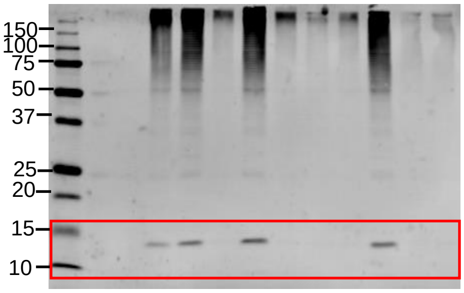

Extended Fig.9b
IB:HA (middle panel)

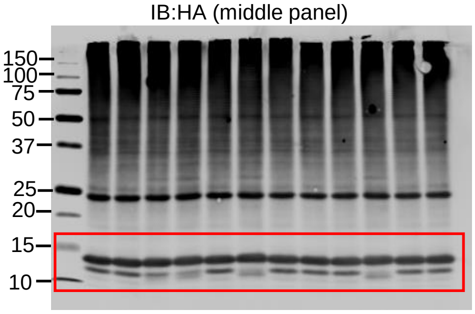

Extended Fig.9b
IB:His (lower panel)

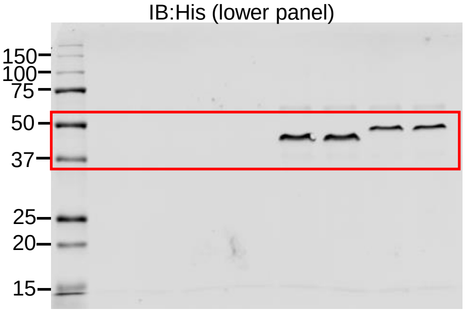

Extended Fig.9b
IB:ICDH (lower panel)

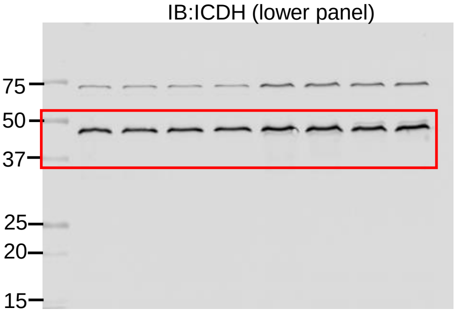

Supplement: Supplementary file 6 — Source Data [file 41467_2024_50311_MOESM6_ESM.zip › Source Data.pdf]
